# A Concise Review on the Role of Endoplasmic Reticulum Stress in the Development of Autoimmunity in Vitiligo Pathogenesis

**DOI:** 10.3389/fimmu.2020.624566

**Published:** 2021-02-04

**Authors:** Shahnawaz D. Jadeja, Jay M. Mayatra, Jayvadan Vaishnav, Nirali Shukla, Rasheedunnisa Begum

**Affiliations:** Department of Biochemistry, Faculty of Science, The Maharaja Sayajirao University of Baroda, Vadodara, India

**Keywords:** endoplasmic reticulum, unfolded protein response, vitiligo, melanocytes, autoimmunity

## Abstract

Vitiligo is characterized by circumscribed depigmented macules in the skin resulting due to the autoimmune destruction of melanocytes from the epidermis. Both humoral as well as cell-mediated autoimmune responses are involved in melanocyte destruction. Several studies including ours have established that oxidative stress is involved in vitiligo onset, while autoimmunity contributes to the disease progression. However, the underlying mechanism involved in programing the onset and progression of the disease remains a conundrum. Based on several direct and indirect evidences, we suggested that endoplasmic reticulum (ER) stress might act as a connecting link between oxidative stress and autoimmunity in vitiligo pathogenesis. Oxidative stress disrupts cellular redox potential that extends to the ER causing the accumulation of misfolded proteins, which activates the unfolded protein response (UPR). The primary aim of UPR is to resolve the stress and restore cellular homeostasis for cell survival. Growing evidences suggest a vital role of UPR in immune regulation. Moreover, defective UPR has been implicated in the development of autoimmunity in several autoimmune disorders. ER stress-activated UPR plays an essential role in the regulation and maintenance of innate as well as adaptive immunity, and a defective UPR may result in systemic/tissue level/organ-specific autoimmunity. This review emphasizes on understanding the role of ER stress-induced UPR in the development of systemic and tissue level autoimmunity in vitiligo pathogenesis and its therapeutics.

## Introduction

Extensive research over the years established that a complex interaction between genetic, environmental, biochemical, and immunological factors collectively generate a microenvironment favoring melanocyte loss in vitiligo (1–3). The complex genetics of vitiligo involves multiple susceptibility loci, incomplete penetrance, and genetic heterogeneity with gene-gene and gene-environment interactions and altered miRNA expression (**Table S1**) (4–6). Accumulation of oxidative stress due to defective recycling of tetrahydrobiopterin, mitochondrial impairment (**Table S2**), and compromised antioxidant system are reported in vitiligo patients (7–11). This accumulated oxidative stress might result in DNA damage, lipid and protein peroxidation, neoantigen formation, and may affect normal melanogenesis in melanocytes (12). Moreover, both humoral and cellular autoimmunity, altered CD4^+^/CD8^+^ T cell ratio, decreased regulatory T cells (Tregs) function, presence of autoreactive anti-melanocyte CD8^+^ T cells in both blood and skin, as well as imbalance of pro- and anti-inflammatory cytokine levels are reported to be involved in vitiligo pathogenesis (2, 13–20). Our extensive population based studies indicated impeded redox and immune homeostasis in the skin and blood of vitiligo patients from Gujarat population (2, 14, 17, 21–41). Hence, based on our observations, we proposed that oxidative stress triggers vitiligo onset, while autoimmunity contributes to the disease progression (2). Despite extensive research, the exact mechanism which connects the triggering factors with the disease progression is still obscure. Investigating the connecting link between the factors involved in onset and progression of vitiligo may enhance our understanding of its pathomechanisms and thereby open new avenues for development of novel therapeutic strategies.

It has been reported that melanocytes from vitiligo patients had dilated endoplasmic reticulum (ER) as compared to healthy melanocytes (42). The accumulation of misfolded proteins in the ER lumen and its dilation are the characteristics of ER stress. Excessive load of protein folding in ER may also generate oxidative stress (43). Several studies suggested the generation of ROS during normal protein folding process in ER and oxidation of ER proteins under oxidative stress led to the accumulation of misfolded proteins (44, 45). Interestingly, vitiligo patients are reported to have significantly elevated homocysteine levels which may induce oxidative stress, ER stress, and expression of pro-inflammatory cytokines (28, 46, 47). Unfolded protein response (UPR) upon ER stress is also known to regulate the innate immune response in different ways (48). Based on several direct and indirect evidences, earlier we speculated that ER stress could be a major link between oxidative stress and autoimmunity, which might play a key role in the onset and exacerbation of vitiligo (49). In this review, we will emphasize on the potential role of ER stress in the development of autoimmune/inflammatory responses in vitiligo.

## Understanding ER Stress-Induced UPR

**Figure 1:**
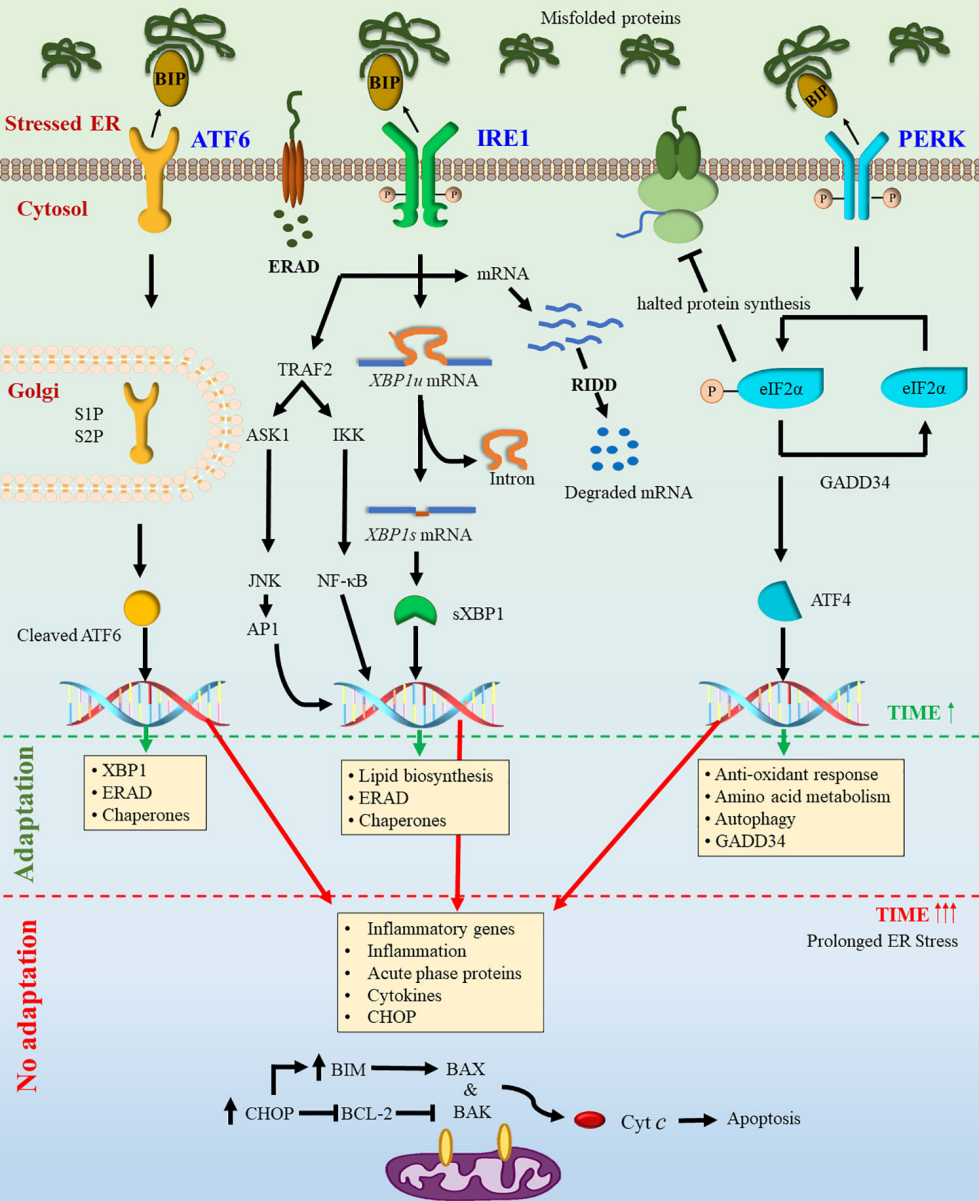
Activation of unfolded protein response. Due to stress conditions, unfolded protein levels increase in the ER lumen. The dissociation of GRP78 from transmembrane sensors PERK, ATF6, and IRE1 leads to the activation of UPR signaling. Activation of IRE1 and PERK results in their oligomerization and transphosphorylation. Active IRE1 triggers the unconventional splicing of *XBP1u* mRNA resulting in the translation of an active transcription factor sXBP1. The active IRE1 can also interact with JNK and TRAF2 and thereby activating downstream signaling. The activation of ATF6 leads to its translocation to the Golgi and its proteolytic cleavage, resulting in a transcriptionally active form. Activation of PERK triggers phosphorylation of elF2α leading to global translational attenuation and favoring translation of ATF4. Activation of all three pathways activate downstream transcriptional machinery resulting in expression of target genes to overcome the stress conditions. Persistent and excessive ER stress may lead to activation of mitochondria mediated cell death pathway.

The ER is an active intracellular organelle with different functions like protein folding and maturation within the eukaryotic cell, essential for cellular homeostasis, proteostasis, cellular development, and stress responsiveness (50). Aberrations in protein folding may result in an imbalance leading to the accumulation of misfolded proteins in the ER, which is known as ER stress. To combat ER stress, the cell activates UPR which may alleviate ER stress through global translation attenuation, induction of chaperones, degradation of misfolded proteins by ER-associated degradation (ERAD), and apoptosis (51). The accumulation of misfolded proteins increases the production of BiP/GRP78 (78-kDa glucose-regulated protein) (52). GRP78 forms dynamic stability between the nascent polypeptides (unfolded proteins) and intra-luminal domains of the three ER stress sensors *viz.* inositol-requiring enzyme 1α (IRE1α), PKR like endoplasmic reticulum kinase (PERK), and activating transcription factor 6 (ATF6) (53–56). In non-stress conditions, all three sensors are primarily bound with GRP78, which helps to maintain its inactive state. The fate of the stressed cell towards survival or death depends upon the interplay among these three major arms of the UPR signaling pathways (57, 58) (**Figure 1**).

## Role of UPR in Inflammation and Immune Regulation

The UPR has emerged as a hallmark of several diseases including inflammatory bowel disease, arthritis, neurodegenerative diseases, diabetes mellitus, stroke, and cancer (58–60). UPR plays a vital role in inflammation, mainly regulated by nuclear factor kappaB (NF-κB) and activator protein 1 (AP-1) (61–63). NF-κB regulates the expression of various genes including those encoding cytokines, chemokines, and *also* participates in inflammasome regulation. All three pathways can activate NF-κB independently, but IRE1α plays an essential role in inflammatory pathways (64). IRE1 interaction with TRAF2 (TNF Receptor Associated Factor 2) in response to ER stress leads to the recruitment of IκB kinase (IKK) which phosphorylates and subsequently degrades IκB (65), thereby activating NF-κB. PERK-eIF2α signaling pathway halts overall protein synthesis. Thus, NF-κB to IκB ratio in cell increases due to IκB’s shorter half-life than NF-κB, which subsequently favors NF-κB dependent transcription (66, 67). Activated IRE1 also interacts with TRAF2 and ASK1 that further activates the JNK in addition to the activation of NF-κB and leading to the AP1 activation (68–70). Genes transcribed by AP1 include cytokines such as tumor necrosis factor (TNF), keratinocyte growth factor (KGF), granulocyte-macrophage colony-stimulating factor (GM-CSF), IL8, IL-1 receptor antagonists, and fibroblast growth factor receptor 1, implying that AP1 also plays a crucial role in the regulation of cytokine receptors (71).

All three major arms of UPR *viz.* PERK, ATF6, and IRE1 have a central role in immune regulation. In PERK signaling, ATF4 activates *IL6* transcription in macrophages (72). Further, phosphorylation of eIF2α upon activation of PERK signaling allows selective translation of mRNAs bearing upstream open reading frames (uORFs) in their 5′-untranslated regions (5′-UTR), which might act as novel antigens for MHC-I presentation (73). IRE1α undergoes phosphorylation by signals downstream to Toll-like receptors (TLRs). Phosphorylated IRE1α induces unspliced *XBP1u* mRNA splicing resulting in an active transcription factor, spliced XBP1 (XBP1s), which activates the production of pro-inflammatory cytokines in macrophages (74). IRE1α/XBP1s also contributes to homeostasis and survival of CD8α^+^ conventional dendritic cells (DCs) (75). Furthermore, it has also been reported that XBP1s may regulate the expression of *TNF* and *IL6* in macrophages (74). Notably, transcriptional targets of ATF6 include XBP1 and thus ATF6 is also recognized as a regulator of the IRE1/XBP1 axis (76–78). Interestingly, it has been reported that cleaved ATF6 can act as an enhancer and increase the CREBH-mediated (cAMP response element-binding protein H) acute inflammatory response, indicating a link between ATF6 and inflammation (79).

## Implications of Localized and Peripheral ER Stress in Vitiligo

### ER Stress in the Skin Microenvironment

In the skin, ER stress may be induced by various endogenous as well as exogenous stressors such as UV irradiation, trauma, and chemical stressors (**Table S3**). Interestingly, chemical stressors including phenolic derivatives such as rhododendrol, hydroquinone, MBEH (mono benzyl ether of hydroquinone), and 4-TBP (4-tertiary butyl phenol) present in the cosmetic skin whitening agents have been identified to induce UPR mediated melanocyte death (80–84). Importantly, physiological ER stress is required for the maintenance of normal biological functions including keratinocyte differentiation in the skin (85). ER stress-signaled UPR was found to be activated during epidermal keratinocyte differentiation (85–87). Expression of UPR activation markers such as sXBP1, CHOP, and GRP78 is increased in the undifferentiated/proliferative stage of keratinocytes during their differentiation (85, 88). Furthermore, CD8^+^ T cells are found to be essential effectors of melanocyte destruction in vitiligo patients (89, 90). The recruitment of CD8^+^ T cells to skin lesions is carried out by the IFN-γ-mediated T cell chemokine receptor, C-X-C motif chemokine receptor 3 (CXCR3), and its ligands CXCL9, CXCL10, and CXCL11, which are found to be abundant in skin biopsy specimens from vitiligo patients (91). The blockade of this pathway mitigated the vitiligo in mice as well as in human subjects (92, 93). IRE1α/sXBP1 signaling in stressed keratinocytes augmented the levels of CXCL16, which is involved in CD8^+^ T cell recruitment to skin lesions (94).

### ER Stress in Peripheral System

Peripheral blood mononuclear cells (PBMCs) play a critical role in immune response, metabolism, and communication with other cells. PBMCs of vitiligo patients were reported to have metabolic deregulations and oxidative stress, similar to those found in melanocytes and the lesional epidermis (95–97). Histological studies have demonstrated that infiltration of CD8^+^ T cells occurs surrounding the vitiligo lesions (98–101). Hence, the role of ER stress in the regulation of the peripheral immune system may be interesting in understanding vitiligo pathogenesis. The UPR signaling is involved in the differentiation, proliferation, and homeostasis of both B and T cells. In the presence of a differentiation stimulus, both B and T cells increase GRP78 protein levels, initiate *XBP1* splicing, and induce ATF6 signaling (102–105). The inhibition of GRP78, ATF6, or XBP1 greatly reduces plasma cell differentiation and their efficacy upon maturation (102, 106). Cell fate determines whether UPR signaling is maintained for example, early B cells exhibit active UPR signaling, but it is absent in mature B cells. Similarly, CD4^−^/CD8^−^ progenitor T cells do not exhibit UPR, but greatly increase UPR during maturation as CD4^+^/CD8^+^ T cells. Upon differentiation to CD4^+^ T cells, the UPR is once again repressed (103). CD8^+^ T cells play a major role in anti-melanocyte autoimmunity in vitiligo. Infection of mice with lymphocytic choriomeningitis virus (LCMV) resulted in the upregulation of spliced and unspliced XBP1 that further enhanced differentiation of CD8^+^ T cells (104). ER stress chaperone, GRP78 also plays an essential role in the regulation of granzyme B in CD8^+^ T cells and CD8^+^ intraepithelial lymphocytes. CD8^+^ T cells of heterozygous GRP78 mouse model had reduced granzyme B secretion and cytotoxicity. This granzyme B deficiency was due to a reduction in IL-2 mediated proliferation, as exogenous IL-2 helped to partially restore granzyme B expression (107).

ER stress is also implicated in the regulation of Treg cells. Human Treg clones had elevated IL-10 production when treated with thapsigargin, an activator of ER stress and UPR, in an eIF2α phosphorylation-dependent manner (108). Loss of ATF4 led to a modest increase in *FOXP3* mRNA expression in mouse CD4^+^ cells differentiated under T regulatory conditions in a high oxidizing environment (109). Recently, decreased levels of NFATs and FOXP3 are reported in Tregs of generalized vitiligo patients which may impair Treg cell function along with reduced IL10 and CTLA4 levels (18–20).

## Plausible Involvement of ER Stress in Vitiligo Autoimmunity

The ER stress may contribute to the development of autoimmunity through the recognition of misfolded proteins by autoreactive immune cells. Release of neo-autoantigens and UPR-related autoantigens by stressed cells, subsequently provoke autoimmunity. ER stress may indirectly contribute to autoimmunity through impairment of immune-tolerance mechanisms in cells with an abnormal UPR and conferring resistance to UPR mediated apoptosis in autoreactive cells by upregulating ERAD-associated proteins (48). Under certain pathophysiological conditions, several ER chaperones are translocated to the cell surface or released in extracellular space, which may serve as damage-associated molecular patterns (DAMPS) and attract the innate immune system to target “abnormal” cells for phagocytosis leading to subsequent activation of adaptive immunity (110). These phenomena have been established in various autoimmune disorders such as type I diabetes (T1D), rheumatoid arthritis (RA), systemic lupus erythematosus (SLE) (111, 112). Interestingly, one of the essential ER chaperones, GRP78 has immunomodulatory functions upon cell surface translocation. Vig et al. (112) have demonstrated that sGRP78 serves as a pro-apoptotic signaling receptor in beta cells and postulated that inflamed beta cells set up a self-destructing feedback loop through the combined surface translocation and secretion of GRP78. These findings suggest an important role of surface translocated GRP78 in autoimmune destruction of target cells. Though the role of sGRP78 is not yet established in melanocyte destruction, a few studies on other chaperones have encouraged researchers to hypothesize its role in melanocyte destruction in vitiligo. Kroll et al. (113) have observed that 4-tertiary butyl-phenol (4-TBP) induced expression and release of HSP70 by PIG3V melanocytes (immortalized vitiligo melanocytes). Further, it induced expression of tumor necrosis factor-related apoptosis-inducing ligand (TRAIL) on the membrane and activated DC effector functions towards the stressed melanocytes. Interestingly, they observed increased expression of TRAIL and CD11c+ dendritic cell infiltration in the perilesional skin of vitiligo patients. This suggested that HSP70 release by stressed melanocytes may facilitate DC activation leading to melanocyte destruction in vitiligo (113). In another exciting study, Zhang et al. have reported oxidative stress-induced translocation of calreticulin (CRT) on melanocyte surface (114). They observed that CRT surface translocation (sCRT) on melanocytes induced expression of pro-inflammatory cytokines such as IL-6 and TNF-a by human PBMCs *in vitro*. Elevated sCRT was concordant with decreased membrane CD47 expression; CD47 acts as a “don’t eat me” signal in contrast to “eat me” signal of CRT, resulting in immunogenic cell death (114, 115). Moreover, a positive correlation of plasma CRT levels was observed with the area affected and the activity of the disease in vitiligo patients suggesting CRT’s role in vitiligo pathogenesis (114). These studies led us to postulate the potential role of ER stress response proteins in the initial development of autoimmune response against stressed melanocytes.

## Future Prospects and Translational Relevance of ER Stress in Vitiligo

As per the recent understanding, it is clear that the ER stress is at the verge of oxidative stress and inflammatory/immunoregulatory response in the cell, making it an ideal therapeutic target. However, the core UPR signaling involved in melanocyte biology and vitiligo pathomechanism is not much explored. A few studies demonstrate that therapeutic agents modulating ER stress can be promising for vitiligo treatment. Zhu et al. (116) have reported that Baicalin attenuated the progression and reduced the area of depigmentation in the C57BL/6 mouse model of vitiligo. Furthermore, they observed that Baicalin stimulated the proliferation of melanocytes in depigmented skin, which further led to a decrease in CD8^+^ T cell infiltration and the expression of CXCL10 and CXCR3 in mice skin. Interestingly, they also observed significantly decreased levels of IL-6, TNF-α, IFN-γ, and IL-13 in sera of vitiligo mice models (116). Baicalin is an active ingredient of *S. baicalensis*, which is reported to protect cardio-myocytes and chondrocytes from ER stress-induced apoptosis (117, 118). Bilobalide is one of the active components of *G. biloba* extract. Lu et al. reported that pre-treatment with bilobalide could protect melanocytes from oxidative damage by inhibiting H_2_O_2_ induced cytotoxicity. It also inhibited eIF2α phosphorylation and downregulated CHOP expression (119). However, the exact mechanism of ER stress modulation by these herbal extracts is not clear. Apart from these, therapeutic strategies aiming to improve protein-folding capacity during ER stress might also be promising. Chemical chaperones such as Tauro-ursodeoxycholic acid (TUDCA) and 4−phenyl butyrate (PBA) can improve protein folding in the ER. Success in the alleviation of ER stress-induced hyperglycemia, restoration of insulin sensitivity, and fatty liver disease amelioration was observed upon TUDCA and 4-PBA treatments in obese mice (120). Cao et al. have reported that TUDCA and 4-PBA decreased ER stress in the intestinal epithelium leading to reduced dextran sodium sulfate (DSS) induced colitis severity (121). Moreover, it was found that 4-PBA leads to a decrease in lipopolysaccharide (LPS)-induced lung inflammation through modulating ER stress, NF−κB, and hypoxia-inducing factor 1α (HIF1α) signaling (122). Nevertheless, further studies to understand the molecular mechanism of ER stress signaling in melanocytes, neighboring keratinocytes, and circulatory as well as infiltrated immune cells are warranted for the development of novel targeted and personalized ER stress modulating therapeutics for vitiligo.

## Conclusions

**Figure 2:**
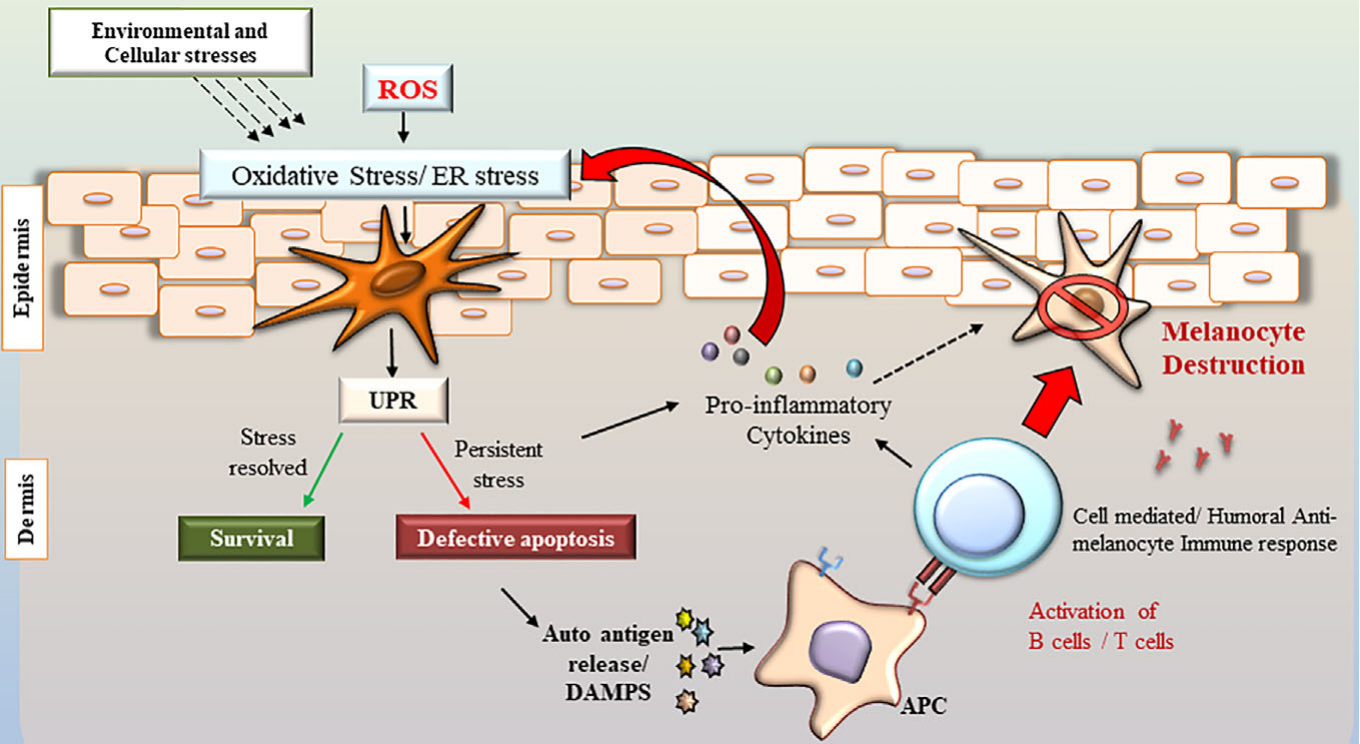
Role of ER stress, oxidative stress, inflammation, and autoimmunity in vitiligo pathogenesis. Various exogenous and endogenous stressors in the skin result in oxidative stress and ER stress. ER stress activates the UPR signaling to resolve the stress. However, prolonged ER stress and defective UPR may lead to activation of inflammatory transcriptional program and release of proinflammatory cytokines, which generates further ER stress and oxidative stress. Further, the defective apoptosis of melanocytes might result in the release of misfolded/unfolded proteins that can potentially act as autoantigens and might be identified as damage-associated molecular patterns by the immune cells. The antigen presenting cells (APC) may process and present the altered proteins/peptides generating novel epitopes, which in turn will activate target B and T cells, resulting in an anti-melanocyte autoimmune response.

Over the decades, the role of UPR in the pathogenesis of various autoimmune disorders is well established. However, its role in anti-melanocyte autoimmunity in vitiligo is yet to be unraveled. Although extensive research has been done to decipher the conundrum of the underlying molecular mechanisms of melanocyte destruction, the role of UPR in vitiligo still remains an enigma. Several studies have uncovered essential direct and indirect mechanistic links that established cross-talk among oxidative stress, ER stress, and autoimmunity, which appears to be crucial in vitiligo pathogenesis (**Figure 2**). A wide range of studies has demonstrated that ER stress-activated UPR plays an essential role in the regulation and maintenance of innate as well as adaptive immunity. Though the role of ER stress in affecting immunity at systemic as well as tissue level is not well understood, a defective UPR might result in organ-specific autoimmunity. Since the immune response is a multi-step process, depending on the microenvironment of the cell, UPR can promote cell survival or death. This review suggests that the UPR is orchestrating the cell fate differently in the active participating immune cells and the target melanocytes. The genetic predisposition and the microenvironment of the target tissue play a major role in deciding the cell fate. Thus, further studies deciphering the tissue/cell type-specific UPR and developing UPR modulating strategies accordingly are warranted. Future research work in this direction will be promising in the development of novel immunotherapeutics for vitiligo.

## Author Contributions

SJ and RB developed the concept. SJ, JM, JV, and NS performed a literature survey contributed to manuscript writing. RB contributed to the critical revision and approval of the article. All authors contributed to the article and approved the submitted version.

## Funding

RB thanks financial support from the Science & Engineering Research Board (SERB), Government of India (Grant No. EMR/2016/001565) and SJ thanks the University Grants Commission (UGC) for Senior Research Fellowship.

## Conflict of Interest

The authors declare that the research was conducted in the absence of any commercial or financial relationships that could be construed as a potential conflict of interest.
