# Supplementary material for: A Concise Review on the Role of Endoplasmic Reticulum Stress in the Development of Autoimmunity in Vitiligo Pathogenesis
Source: Front Immunol. 2021 Feb 4;11:624566. doi: 10.3389/fimmu.2020.624566 (PMC7890234; doi:10.3389/fimmu.2020.624566)
Supplement: Supplementary file 1 [file DataSheet_1.doc]

**Table S1:** Key miRNAs involved in ER stress, oxidative stress and immune regulation

| **Sr. No** | **miRNA** | **Involvement in regulation of**  **ER stress/ oxidative stress/**  **immunity** | **Expression in vitiligo** | **References** |
| --- | --- | --- | --- | --- |
|
| **1** | miRNA-25-5p | Oxidative stress | Increase | Shi *et al.,* 2016 |
| **2** | miRNA-423-5p | Oxidative stress | Increase | Zhang Y, 2011 |
| **3** | miRNA-211-5p | Oxidative stress,  ER stress and  Immunity | Decrease | Shao *et al.*, 2017; Spiegelman *et al.,* 2017; Sun *et al.,* 2020; Mansuri *et al.,* 2016 |
| **4** | miRNA-1 | Oxidative stress | Increase | Mansuri *et al.,* 2016; Xu et al., 2007 |
| **5** | miRNA-328 | Immunity | Decrease | Mansuri *et al.,* 2016 |
| **6** | miRNA577 | Immunity | Increase | Mansuri *et al.,* 2016 |

**Table S2:** Oxidative stress induced mitochondrial alterations in vitiligo

| **Sr. No** | **Mitochondrial alterations in vitiligo** | **Levels** | **References** |
| --- | --- | --- | --- |
|
| **1** | Mitochondrial membrane potential | Decrease | Dell’Anna et al., 2003 |
| **2** | Mitochondrial NAD-dependent deacetylase sirtuin-3 (SIRT3) | Decrease | Yi et al., 2019 |
| **3** | Malate dehydrogenase (MDH) | Increase | Dell’Anna et al., 2003 |
| **4** | Cardiolipin (CL) | Decrease | Dell’Anna et al., 2010 |
| **5** | Peroxisome proliferator-activated receptor gamma coactivator 1-alpha (PGC1-α) | Increase | Sahoo et al., 2017 |
| **6** | p53 | Increase | Bellei et al., 2013 |
| **7** | Caspase 3 | Increase | Prignano et al., 2009 |
| **8** | Caspase 8 | Increase |
| **9** | Caspase 9 | Increase |
| **10** | Adenosine triphosphate (ATP) | Decrease | Dell’Anna et al., 2017 |

**Table S3:** Potential ER stressors in vitiligo

| **Potential ER stressors in vitiligo** | |
| --- | --- |
| **Endogenous stressors** | **Exogenous stressors** |
| H_2_O_2_ (Schallreuter et al. 1999) | Ultraviolet light (Xie et al. 2016) |
| Homocysteine (Anbar et al., 2016; Jadeja et al. 2018) | Ionizing radiation (Trouba et al. 2002) |
| Pro-inflammatory cytokines (Singh et al., 2018) | Xenobiotics (Minashkin et al. 2013) |
| Nitric oxide/ Peroxynitrite (Salem et al. 2009; Schallreuter, 1999) | Mono benzyl ether of hydroquinone (MBEH)  (Nordlund 2007; Harris 2017) |
| Catecholamines (Morrone et al. 1992) | 4-tertiary butyl phenol (4-TBP) (Nordlund 2007; Harris 2017) |
| Tetrahydrobiopterin (Schallreuter et al. 1998) | Rhododendrol (Sasaki et al. 2014) |

**References:**

1. Schallreuter KU, Moore J, Wood JM, et al (1999) In vivo and in vitro evidence for hydrogen peroxide (H2O2) accumulation in the epidermis of patients with vitiligo and its successful removal by a UVB-activated pseudocatalase. In: Journal of Investigative Dermatology Symposium Proceedings. 4(1):91-96.
2. Schallreuter KU (1999) A review of recent advances on the regulation of pigmentation in the human epidermis. Cell Mol Biol (Noisy-le-grand) 45:943–949.
3. Schallreuter KU, Zschiesche M, Moore J, et al (1998) In VivoEvidence for Compromised Phenylalanine Metabolism in Vitiligo. Biochem Biophys Res Commun 243:395–399. https://doi.org/10.1006/bbrc.1997.8107.
4. Anbar T, Zuel-Fakkar NM, Matta MF, Arbab MMI (2016) Elevated homocysteine levels in suction-induced blister fluid of active vitiligo lesions. Eur J Dermatology 26:64–67. https://doi.org/10.1684/ejd.2015.2682
5. Jadeja SD, Mansuri MS, Singh M, et al (2018) Association of elevated homocysteine levels and Methylenetetrahydrofolate reductase (MTHFR) 1298 A>C polymorphism with Vitiligo susceptibility in Gujarat. J Dermatol Sci 90:112–122. https://doi.org/10.1016/j.jdermsci.2018.01.003
6. Singh M, Kotnis A, Jadeja SD, et al (2018) Cytokines: the yin and yang of vitiligo pathogenesis. Expert Rev Clin Immunol 1744666X.2019.1550358. https://doi.org/10.1080/1744666X.2019.1550358
7. Salem MMAEL, Shalbaf M, Gibbons NCJ, et al (2009) Enhanced DNA binding capacity on up‐regulated epidermal wild‐type p53 in vitiligo by H 2 O 2 ‐mediated oxidation: a possible repair mechanism for DNA damage . FASEB J 23:3790–3807. https://doi.org/10.1096/fj.09-132621
8. Morrone A, Picardo M, Luca C De, et al (1992) Catecholamines and Vitiligo. Pigment Cell Res 5:65–69. https://doi.org/10.1111/j.1600-0749.1992.tb00003.x
9. Xie H, Zhou F, Liu L, et al (2016) Vitiligo: How do oxidative stress-induced autoantigens trigger autoimmunity? J Dermatol Sci 81:3–9. https://doi.org/10.1016/j.jdermsci.2015.09.003
10. Trouba KJ, Hamadeh HK, Amin RP, Germolec DR (2002) Oxidative stress and its role in skin disease. Antioxidants Redox Signal. 4:665–673
11. Minashkin MM, Salnikova LE, Lomonosov KM, et al (2013) Possible contribution of GSTP1 and other xenobiotic metabolizing genes to vitiligo susceptibility. Arch Dermatol Res 305:233–239. https://doi.org/10.1007/s00403-012-1301-x
12. Nordlund J (2007) Vitiligo : a Monograph on the Basic and Clinical Science. John Wiley & Sons
13. Harris JE (2017) Chemical-Induced Vitiligo. Dermatol Clin 35:151–161. https://doi.org/10.1016/j.det.2016.11.006.
14. Sasaki M, Kondo M, Sato K, et al (2014) Rhododendrol, a depigmentation-inducing phenolic compound, exerts melanocyte cytotoxicity via a tyrosinase-dependent mechanism. Pigment Cell Melanoma Res 27:754–763. https://doi.org/10.1111/pcmr.12269.
15. Bellei B, Pitisci A, Ottaviani M, Ludovici M, Cota C, Luzi F, Picardo M. (2013). Vitiligo: A Possible Model of Degenerative Diseases. *PLoS ONE*, *8*(3), 1–12. https://doi.org/10.1371/journal.pone.0059782
16. Dell’Anna, ML, Ottaviani M, Bellei B, Albanesi V, Cossarizza A, Rossi L, & Picardo M. (2010). Membrane lipid defects are responsible for the generation of reactive oxygen species in peripheral blood mononuclear cells from vitiligo patients. *Journal of Cellular Physiology*, *223*(1), 187–193. https://doi.org/10.1002/jcp.22027
17. Dell’Anna, ML, Ottaviani M, Kovacs D, Mirabilii S, Brown DA, Cota C, Picardo M. (2017). Energetic mitochondrial failing in vitiligo and possible rescue by cardiolipin. *Scientific Reports*, *7*(1), 13663. https://doi.org/10.1038/s41598-017-13961-5
18. Dell’Anna, ML, Urbanelli S, Mastrofrancesco A, Camera E, Iacovelli P, Leone G, Picardo M. (2003). Alterations of mitochondria in peripheral blood mononuclear cells of vitiligo patients. *Pigment Cell Research*, *16*(5), 553–559. https://doi.org/10.1034/j.1600-0749.2003.00087.x
19. Prignano F, Pescitelli L, Becatti M, Di Gennaro P, Fiorillo C, Taddei N, & Lotti T. (2009). Ultrastructural and functional alterations of mitochondria in perilesional vitiligo skin. *Journal of Dermatological Science*, *54*(3), 157–167. https://doi.org/10.1016/j.jdermsci.2009.02.004
20. Sahoo A, Lee B, Boniface K, Seneschal J, Sahoo SK, Seki T, Perera RJ. (2017). MicroRNA-211 Regulates Oxidative Phosphorylation and Energy Metabolism in Human Vitiligo. *The Journal of Investigative Dermatology*, *137*(9), 1965–1974. https://doi.org/10.1016/j.jid.2017.04.025
21. Yi X, Guo W, Shi Q, Yang Y, Zhang W, Chen X, Li C. (2019). SIRT3-Dependent Mitochondrial Dynamics Remodeling Contributes to Oxidative Stress-Induced Melanocyte Degeneration in Vitiligo. *Theranostics*, *9*(6), 1614–1633. https://doi.org/10.7150/thno.30398
22. Shi Q, Zhang W, Guo S, Jian Z, Li S, Li K et al (2016) Oxidative stress-induced overexpression of miR-25: the mechanism underlying the degeneration of melanocytes in vitiligo. Cell Death Differ. 23(3):496–508.
23. Zhang Y (2011) The mechanism research of miR-423-5p regulated human melanocyte oxidative stress injury induced by hydrogen peroxide [Ph.D]. Fourth Military Medical University
24. Sahoo A, Lee B, Boniface K, Seneschal J, Sahoo SK, Seki T et al (2017) MicroRNA-211 regulates oxidative phosphorylation and energy metabolism in human vitiligo. J Invest Dermatol 137(9):1965–1974.
25. Spiegelman VS, Elcheva IA, Metabo-miR (2017) miR-211 regulates mitochondrial energy metabolism in vitiligo. J Invest Dermatol 137(9):1828–1830.
26. Sun X, Wang T, Huang B, Ruan G, & Xu A. (2020). ΜicroRNA‑421 participates in vitiligo development through regulating human melanocyte survival by targeting receptor‑interacting serine/threonine kinase 1. Molecular Medicine Reports, 21(2), 858-866.
27. Xu C, Lu Y, Pan Z et al. The muscle-specific microRNAs miR-1 and miR-133 produce opposing effects on apoptosis by targeting HSP60, HSP70 and caspase-9 in cardiomyocytes. J Cell Sci 2007; 120: 3045-52.
28. Mansuri MS, Singh M, & Begum R. (2016). miRNA signatures and transcriptional regulation of their target genes in vitiligo. Journal of dermatological science, 84(1), 50-58.
